# Green transformations in Vietnam's energy sector

**DOI:** 10.1002/app5.251

**Published:** 2018-08-09

**Authors:** Frauke Urban, Giuseppina Siciliano, Linda Wallbott, Markus Lederer, Anh Dang Nguyen

**Affiliations:** ^1^ Centre for Development, Environment and Policy CeDEP, SOAS University of London 36 Gordon Square London WC1H 0PD UK; ^2^ Institute of Political Science Technical University Darmstadt Dolivostraße15 Darmstadt 64293 Germany; ^3^ Vietnamese Academy of Social Sciences 1 Liễu Giai, Ba Đình Hà Nội Vietnam

**Keywords:** climate, energy justice, hydro, solar, wind

## Abstract

Vietnam has experienced rapid economic growth over the past few decades, as well as growing environmental pressures. The country is therefore pursuing strategies for green transformations, which are the processes of restructuring to bring economies and societies within the planetary boundaries. This article addresses the opportunities, barriers, and trade‐offs for green transformations in Vietnam's energy sector and examines them from an energy justice perspective. The article draws on in‐depths expert interviews with representatives from government agencies, private firms, academic institutions, and multilateral institutions in Vietnam. The article finds that Vietnam is undergoing efforts to move away from business as usual by promoting renewable energy and energy efficiency, as well as aligning energy and climate plans with national development priorities such as energy security and economic growth. Yet there is a need for more coordinated, integrated approaches and policies that span across the 3 areas that address green transformations in Vietnam: green growth, sustainable development, and climate change. Finally, although key actors seem to be aware and may be critical of major trade‐offs such as land grabs for energy projects, the impacts on affected people need to be better understood and mitigated.

## 1 INTRODUCTION

Vietnam has experienced rapid economic growth over the past two decades, making it one of the strongest and fastest growing economies in Southeast Asia. At the same time, the country has experienced increasing levels of urbanisation and industrialisation as well as high population growth. The poverty headcount declined from nearly 60% to 20% in the past 20 years in Vietnam (World Bank, 2017). Although millions of people have been lifted out of poverty, the recent development trajectory has also resulted in increasing environmental pressures (World Bank, 2017). Over the last two decades, Vietnam has seen a steep increase in CO_2_ emissions that contribute to climate change, a steadily growing energy demand and an increase in other environmental pressures such as air pollution, water pollution, soil pollution, deforestation, natural habitat destruction, and biodiversity loss (World Bank, 2017).

Thus, we need new strategies to “governing the commons” (Ostrom, 1990, 2014) to ensure humanity lives sustainably on planet Earth. And, indeed, options are debated of how to restructure economies and societies in ways that enable environmental sustainability while promoting economic and social development. Suggestions for achieving this include radical economic and societal restructuring through degrowth (Jackson, 2009, 2011). On the contrary, others argue for greening capitalism by pricing nature (Constanza et al., 1998; Spratt, 2015) and increasing investments in green technological innovation (Geels & Schot, 2007; Stern & Rydge, 2014). All of these options offer opportunities but also barriers, particularly for a middle income country such as Vietnam.

What might be required is a relatively radical approach that goes beyond business‐as‐usual scenarios with an added technological fix. Therefore, this article uses the concept of “green transformations” (Lederer, Wallbott, & Bauer, 2018) that are defined as the processes of restructuring, which bring economies and societies within the planetary boundaries (Scoones, Newell, & Leach, 2015). Green transformations can be interpreted as practices of radical economic, societal, and institutional change that transcends sectors and levels (Schmitz & Becker, 2013).

Vietnam is actively pursuing green transformations in the energy sector. This is happening by a recent shift in the country's climate policy focus from adaptation to mitigation. The shift is motivated by multiple domestic policy goals other than mitigation, including economic restructuring, improving energy security, accessing international finance to overcome the phasing‐out of conventional development aid, and accessing climate‐relevant technology. Cobenefits and negative cost‐options, such as achieved by energy efficiency measures, are a major incentive for promoting green transformations (Zimmer, Jakob, & Steckel, 2015).

This article addresses the opportunities, barriers, and trade‐offs for green transformations in Vietnam's energy sector and examines this from an energy justice perspective. We draw on in‐depths expert interviews with representatives from government agencies, private firms, academic institutions, and multilateral institutions in Vietnam. We also evaluated recent energy data for Vietnam.

Section 2 will present the concepts and methods, Section 3 will elaborate the findings, and Section 4 will discuss the findings and policy implications.

## 2 CONCEPTS AND METHODS

The article draws on two key concepts: green transformations and energy justice, which will be elaborated in the following sections.

### 2.1 Green transformations

#### 2.1.1 Green transformations: Different from transitions

This section aims to define the term green transformation. First, we need to define the term “green.” Scoones et al. (2015) broadly defines “green” as symbolising nature or the environment. She divides “green politics” into three categories: “light green” meaning the environment may be protected by market incentives, such as through carbon trading or fiscal incentives for a green economy; “dark green,” which regards the environment as much more vulnerable and applies the precautionary approach for its protection and conservation; and “bright green,” which aspires for an optimistic future based on green technological and social innovation (Lederer et al., 2018; Lederer, Wallbott, & Urban, Forthcoming).

Second, we need to define the term “transformation” and distinguish it from the term “transition.” Here, we refer to Stirling (2015, p. 62) who argues that transitions “are managed under orderly control, through incumbent structures, according to tightly disciplined technical knowledge and innovations.” On the other hand, “transformations imply more diverse, emergent and unruly political alignments, challenging incumbent structures, subject to incommensurable, tacit and embodied social knowledge and innovations.” Hence, we would suggest that transformations are more aimed at reconfiguration of structures, processes, and actor constellations, thereby potentially challenging the polities (institutions), policies (content), and also politics (processes) of a specific system (Lederer et al., 2018; Lederer et al., Forthcoming).

Conclusively, green transformations can be defined as the restructuring of political, social, and economic systems to fit within the planetary boundaries (Scoones et al., 2015). A similarly ambitious understanding is suggested by the Heinrich‐Böll‐Stiftung, (2013 p. 1), which equals green transformations with a “great transformation.” Accordingly, they argue that industrial societies should be transformed into “a climate compatible, resource‐conserving and sustainable world economic order.” That would require far‐reaching and long‐term changes in scientific, technological, social, and political systems and across global, regional, national, and local levels (Lederer et al., 2018; Lederer et al., Forthcoming).

Green transformations are therefore more complex and far‐reaching than transitions. We refer to transformations in plural, rather than transformation in singular as multiple green transformations are needed, such as from high‐carbon to low‐carbon development (Urban & Nordensvärd, 2013), from excessive natural resource depletion to natural resource conservation, and from deforestation to afforestation. It is therefore a holistic and wide‐ranging set of processes (Lederer et al., 2018). At the same time, green transformations have the objective to be socially just, equitable, and balancing the ecological, economic, and social dimensions (WBGU, 2011). Green technologies, such as renewable energy, and market incentives such as carbon markets can be helpful, yet we argue that meaningful green transformations will not evolve based on technological solutions only and that a greening of the economy will not be sufficient. Instead, revolutionary changes are necessary for what Stern and Rydge (2014) describe as a new energy‐industrial revolution.

Scoones et al. (2015) argue that drivers for green transformations stem either from techno‐centric or market‐based innovations or are state‐ or citizen‐led. We suggest that these approaches can broadly be classified as “material”‐centered (techno‐centric and market‐based transformations) and “actor”‐centered (citizen‐led and state‐led transformations). It is unlikely that green transformations will happen in one major strike. Instead, they may be incremental, nonlinear, happening at different scales, for different reasons than just environmental reasons, and driven forward by various actors (Lederer et al., 2018; Lederer et al., Forthcoming).

#### 2.1.2 Green transformations: Different from a Green Economy

In addition, green transformations differ from the concepts of a Green Economy or green growth (Lederer et al., 2018). International organisations such as the United Nations Environmental Program (UNEP) and the World Bank have recently promoted the Green Economy, arguing that it “results in improved human well‐being and social equity, while significantly reducing environmental risks and ecological scarcities. In its simplest expression, a green economy can be thought of as one which is low carbon, resource efficient and socially inclusive” (UNEP, 2012, p. 2).

The concern for environmental, social, and economic sustainability is shared between the Green Economy and green transformations, yet a Green Economy is different in that it strongly focuses on an efficient and functioning economy as a precondition for implementing change. The terminology of the Green Economy implies that it follows the dominant neo‐liberal approach to global governance and favours market‐based instruments (Bernstein, 2002; Crist, 2007). The Green Economy lens might also omit capturing the opportunities and barriers of transformative processes that are broader in scale and scope, and the political and social dynamics that come with it. This is where the terminology “green transformation” goes beyond a functionalist Green Economy approach, albeit green transformations can incorporate elements of the Green Economy to achieve wider goals (Lederer et al., 2018).

### 2.2 Energy justice

“The energy justice framework” defines energy justice “as a global energy system that fairly disseminates both the benefits and costs of energy services, and one that contributes to more representative and impartial energy decision‐making” (Sovacool, Heffron, McCauley, & Goldthau, 2016, p. 4). It examines four key elements of justice, namely, “recognition” of those affected by energy injustices, a fair “distribution” in society of costs and benefits from energy services, a fair “procedure” in decision‐making processes, and “restorative justice,” referring to the actions that need to be taken into account to compensate for the potential adverse effects that can result from energy decisions (Heffron & McCauley, 2017). For energy projects, these restorative actions can include the implementation of Social and Environmental Impact Assessments before energy decisions are made, the application of social safeguards measures, monitoring and evaluation of energy projects, and reducing the impacts after construction and operation. Restorative justice is therefore applied to all phases of an energy project (Siciliano, Urban, Tan‐Mullins, & Mohan, 2018).

The energy justice framework further suggests nine principles to analyse energy systems, such as “availability” (ensuring energy supply is available at adequate quality), “affordability” (access to affordable energy services, especially for the poor), “transparency and accountability” (access to high‐quality information about energy and its impacts, and transparent, accountable, and fair energy decision‐making), “intragenerational and intergenerational equity” (fair access to energy services for present and future generations), “responsibility” (protecting the natural environment and reducing energy‐related environmental adverse effects), “due process” (respecting human rights in energy generation, transmission, supply, and use), “sustainability” (energy resources should not be depleted at an unsustainable rate), “resistance” (opposition to energy injustices), and “intersectionality” (recognition of new modern identities in society and linkages to different forms of injustices) (Siciliano et al., 2018; Sovacool, Burke, Baker, Kotikalapudi, & Wlokas, 2017).

The roots of this framework stem from literature on social justice from a philosophical and ethical perspective. Social justice is therefore based on the idea of a balance between fair allocation of goods and bads (Campbell, 2010; Gordon, 1980; Hinman, 2008). The philosopher John Rawls argues that the primary subject of justice “[…] is the way in which the major social institutions determine the appropriate distribution of the benefits and burdens of social cooperation” (Rawls, 1971 pp. 4 and 11). This emphasises the principles of “procedural justice” (the way in which decision‐making takes into account social justice concerns) and “distributional justice” (the distribution of benefits and burdens; Boström, 2012; Nordensvard, Urban, & Mang, 2015; Schlosberg, 2003; Schlosberg, 2009; Urban, Nordensvard, Siciliano, & Li, 2015). Procedural justice is mainly concerned with the process, fairness, and transparency of decisions (such as participation of the local population in energy projects), inclusiveness in the decision‐making process (such as recognition of the adverse effects on people and the environment), and legitimacy of institutions involved in decision‐making (such as power relations; Paavola & Adger, 2006; Weston, 2008; Schlosberg, 2003, 2009). Distributive environmental justice is mainly concerned about the equal distribution of energy services and its benefits (such as electricity access) as well as its costs (such as emissions) within society (Arnold, 2011; Schlosberg, 2003, 2009; Siciliano et al., 2018).

**Figure 1:**
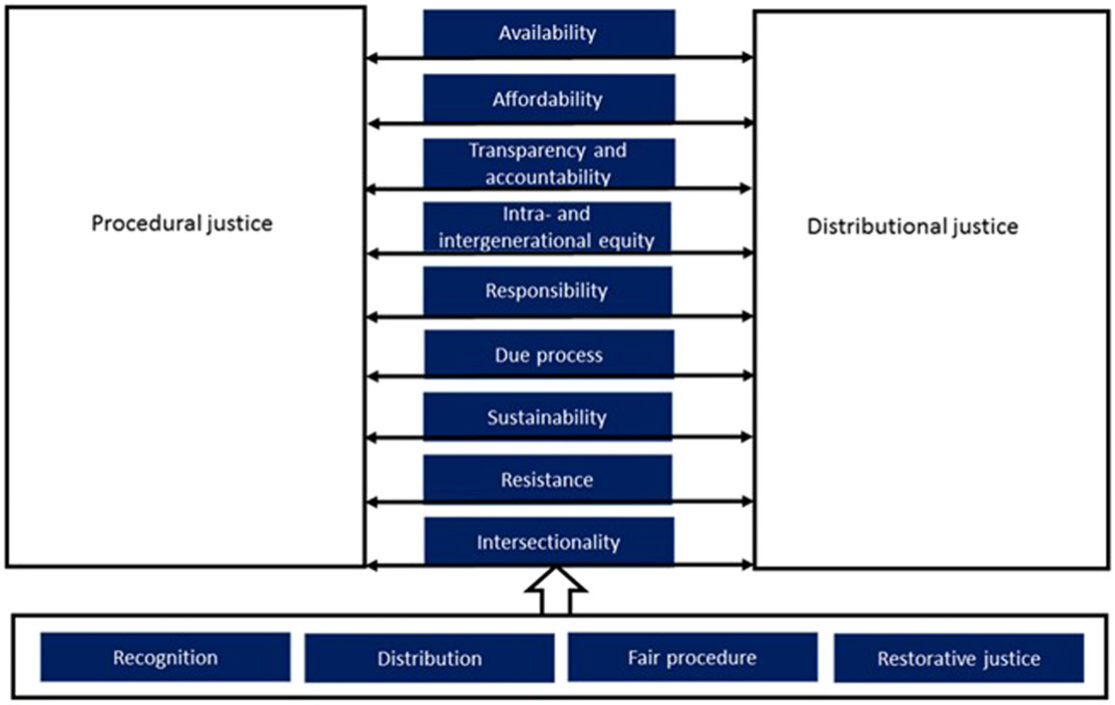
Amended energy justice framework (own compilation on the basis of authors listed above)

Figure 1 indicates the amended energy justice framework that is being used for this study, based on inspirations drawn from Sovacool et al. (2016); Sovacool et al. (2017); Heffron and McCauley (2017); Siciliano et al. (2018); Kirchherr and Charles (2016); Majid Cooke, Nordensvard, Bin Saat, Urban, and Siciliano (2017); Nordensvard et al. (2015); and Urban et al. (2015). Sovacool et al. (2016) argue that energy projects are often placed in a moral vacuum. To make justice concerns central to energy projects, they developed an energy justice framework and applied it to nuclear waste, involuntary resettlement, energy pollution, energy poverty, and climate change by focussing particularly on availability, affordability, due process, transparency and accountability, sustainability, equity, and responsibility. Kirchherr and Charles (2016), Nordensvard et al. (2015), and Majid Cooke et al. (2017) developed different energy justice frameworks for assessing the social justice dimensions of hydropower projects. Nordensvard et al. (2015), Urban et al. (2015), and Majid Cooke et al. (2017) focused particularly on the procedural and distributive justice perspectives. Siciliano et al. (2018) developed an amended energy justice framework that merged the frameworks by Sovacool et al. (2016), Sovacool et al. (2017), and Kirchherr and Charles (2016). This article draws on this framework thinking by these authors, taking into account the nine principles defined by Sovacool et al. (2016) to investigate energy projects, namely, availability, affordability, transparency and accountability, intragenerational and intergenerational, responsibility, due process, sustainability, resistance, and intersectionality. These principles influence procedural and distribute justice on all levels of energy planning (Majid Cooke et al., 2017; Nordensvard et al., 2015). Finally, the four key elements of justice, namely, recognition, fair distribution, fair procedure, and restorative justice (Heffron & McCauley, 2017) influence all of these issues. See Figure 1 for details.

### 2.3 Methodology

Four types of groups were interviewed in the course of primary field work in Vietnam: (a) policymakers from government and bureaucracy, (b) representatives from firms and entrepreneurs, (c) experts from civil society and academia, and (d) representatives from multilateral organisations and donors. We selected the interviewees based on their functions and their role in energy policymaking in Vietnam. All of the interviewees were in senior positions, including directors, vice presidents, and chairmen of governmental think tanks, government departments, university departments, business associations, and senior advisors from multilateral organisations and donors. The interviewing process consisted of semistructured, open questions. A total of 20 interviews were conducted in October 2016 in Hanoi and Lao Cai province. The following institutions were interviewed: Asian Development Bank (ADB), Centre for Eco‐community Development (VAMEC), Coc San hydropower plant operator/InfraCo Asia, Deutsche Gesellschaft für Internationale Zusammenarbeit (GIZ), Development Strategic Institute (DSI), Forestry Service, Global Green Growth Institute (GGGI), Institute of African and Middle East Studies, Institute of Energy Science (Academy of Natural Science and Technology), Institute of Human Geography, Institute of Hydropower and Renewable Energy (IHR), Institute of Policy and Strategy for Agriculture and Rural Development, Lao Cao Authority (Peoples' Committee), Ministry of Environment and Natural Resources (MENR), Ministry of Planning and Investments (MPI), Vietnamese Academy of Social Science–VASS (three interviews with experts), and World Bank. Lao Cai province in Northern Vietnam has been selected as a fieldwork location because it has abundant natural resources that are being exploited for renewable energy development, particularly hydropower and solar energy. It is also a province that is relatively poor compared with other provinces, despite an increasing influx of tourists. The share of ethnic minorities is relatively high in comparison with other provinces in Vietnam, yet many of the ethnic peoples are disproportionally effected by poverty. Green transformations are therefore considered an opportunity for socio‐economic development in Lao Cai.

This primary data were supplemented with secondary data—such as key policy and legislative documents, program and project documents in the field of energy, data on emissions, and energy use from national and international agencies. Data came from two main sources: (a) International databases such as from the International Energy Agency IEA, the World Bank, the Intergovernmental Panel on Climate Change IPCC, and UNEP's Global Environmental Outlook GEO and (b) National databases from relevant ministries and government departments, such as the Ministry of Natural Resources and Environment in Vietnam, the Ministry of Industry and Commerce in Vietnam, and Rural Development in Vietnam. For the energy sector, individual energy producers were also approached regarding data access.

This resulted in the evaluation of both qualitative and the quantitative data. Qualitative data from the interviews and focus group discussions are central to the study. Qualitative data analysis involves categorizing and coding the sources as a means of comparing and contrasting interpretations of events. Materials were stored on digital media and were analysed using narrative analysis rather than conventional “code and retrieve” because the former allows for more layers of embodied meaning to be revealed by including narrative style. In addition, quantitative data such as from the IEA and World Bank databases were used to support the qualitative findings with hard data.

## 3 FINDINGS

### 3.1 Opportunities and barriers for green transformations in Vietnam

Vietnam is actively driving forward policies and actions to adapt to climate change; it is also developing strategies to move towards more low‐carbon, climate‐friendly energy sources such as hydropower, wind, solar, and modern biomass. Yet over 70% of Vietnam's primary energy supply comes from fossil fuels, mainly coal and oil (especially for transport), but increasingly also natural gas (IEA, 2017). Green transformations in Vietnam therefore require a move away from a heavy reliance on fossil fuels to renewable energy, as well as focussing on energy efficiency. Table 1 shows key socio‐economic, energy, and climate indicators for Vietnam.

**Table 1:** Key energy and climate indicators for Vietnam, base line 2015

Key indicators	
Population (millions)	91.71
GDP (billion 2010 USD)	154.51
GDP PPP (billion 2010 USD)	509.26
Energy production (Mtoe)	70.35
Net imports (Mtoe)	5.65
TPES (Mtoe)	73.80
Electricity consumption (TWh)	140.72
CO_2_ emissions (Mt of CO_2_)	168.29
TPES/population (toe/capita)	0.8
TPES/GDP (toe/thousand 2010 USD	0.48
TPES/GDP PPP (toe/thousand 2010 USD)	0.14
Electricity consumption/population (MWh/capita)	1.53
CO_2_/TPES (t CO_2_/toe)	2.28
CO_2_/population (t CO_2_/2010 USD)	1.83
CO_2_/GDP (kg CO_2_/2010 USD)	1.09
CO_2_/GDP PPP(kg CO_2_/2010 USD)	0.33

Data source: IEA (2017)

With regard to opportunities for green transformations, our fieldwork finds that there are three different strategies in Vietnam in relation to green transformations: sustainable development, green growth, and tackling climate change according to the interviewees. The Green Growth Strategy (2012), the Green Growth Action Plan (2014), and the National Strategy on Climate Change (2011) are key policies that aim to enable green transformations in Vietnam. Each strategy has different policies and intervention plans; however, there is a lack of coordination among the agendas for sustainable development and green growth and with the national climate change strategy. Interviewees mention why Vietnam is actively pursuing a climate strategy:

Vietnam is viewed as a high‐risk country for climate change. Typhoons, flooding and other climatic impacts are seen on a daily basis. The plans and policies are driven by the government due to a high risk to climate change. Vietnam has developed an INDC [Intended National Determined Contribution], it aims to reduce its emissions and implement international agreements and commitments. There is also a high government awareness for green growth and sustainable development. (Interview with representative from ADB, October 5, 2016)

The green growth strategy is mainly informed by the Korean GGGI. Korea is also the main partner for the definition of green growth strategies in Cambodia and Laos with financial support, technology transfer, and research.

Conceptually, green growth is mainly driven by the economic sector, whereas sustainable development pays more attention to the social aspects of development. The green growth strategy in Vietnam has three main pillars: (a) reduction of greenhouse gas (GHG) emissions (environment), (b) greening of lifestyles and sustainable consumption (society), (c) clean industrial production (economy). The sustainable development strategy has three pillars too: (a) poverty reduction, (b) education, (c) social welfare.

Through its official policies and programs, the Vietnamese government indicates a serious commitment to the achievement of green transformations. An ad hoc committee at the national level is therefore tasked with the definition and implementation of the National Green Growth Strategy. Moreover, specific measures are under development for the local implementation of green growth and climate change strategies, especially at the provincial level, such as provincial action plans on green growth and for implementing the UNFCCC Paris Agreement.

Our fieldwork reveals that the main barriers for the implementation of green transformation strategies are (a) confusion about the strategies for green growth, sustainable development, and tackling climate change; (b) competing ways of implementing these three strategies as well as competing policies (e.g., policies that favour industrial development and economic growth vs. policies that favour environmental protection vs. policies that favour reduction of GHG emissions); (c) lack of coordination between experts and policies; (d) lack of a policy framework to attract investments in the field of green transformations and (e) the roadmap for introducing renewable energy is very modest and not ambitious enough; and (f) financial restrictions and increasing demand for energy (interviews with representative from ADB and the GGGI in Vietnam, October 5 and 10, 2016).

Interviewees suggest that this could be improved by introducing an institutional reform to coordinate different policies dealing with similar issues, to have better enforcement and monitoring in place for specific issues that span all three strategies, as well as more ambitious renewable energy plans and reducing the reliance on fossil fuels.

### 3.2 Energy issues

#### 3.2.1 Policies and plans for green transformations related to the energy sector

As already highlighted, energy issues are high on Vietnam's transformation agenda and are an important avenue for green transformations. The government issued a number of energy‐related policies to reduce GHG emissions and increase energy efficiency, including the National Target Programme on Energy Efficiency (2006), the Law on the Economical and Efficient use of Energy (2010), and policies on carbon trading (2012; UNFCC, 2015). Renewable energy is actively being promoted by the National Energy Development Strategy (2007) that includes targets for renewable energy for 2020 and a vision for 2050. The Vietnamese government aims to increase the share of renewable energy among electricity generation, excluding hydropower, to 5–8% by 2020, compared with 3.5% in 2010. By 2025, the installed capacity of renewable energy should be 4,050 MW (Dang, 2016).

Prior to the Paris Agreement, Vietnam submitted its Intended National Determined Contribution (INDC) to the United Nations Framework Convention on Climate Change (UNFCCC). The INDC states that Vietnam intends to reduce its total GHG emissions by 8% by 2030 compared with business as usual, and emission intensity reduction will be 20% by 2030 compared with 2010 levels. If bilateral and multilateral financial and technical support will be made available to Vietnam under the Paris Climate Agreement, the target can be increased to 25% GHG emission reductions and 30% emission intensity reduction by 2030 compared with 2010 levels. Energy plays a crucial role in the achievement of these targets (UNFCC, 2015).

Energy issues are on top of the national and international agenda due to the need for energy supplies, large pockets of energy poverty, and the dependence on fossil fuels, particularly coal. The Intergovernmental Panel on Climate Change (IPCC) estimates that about 70% of all GHG emissions worldwide come from energy‐related activities, mainly from fossil fuel combustion for heat supply and electricity generation across all sectors, as well as for transport (IPCC, 2007). In Vietnam, the energy sector is reported to account for about 65% of national GHG emissions (Climatelinks, 2016). The energy supply sector alone contributes to about 35% of global anthropogenic GHG emissions, thereby contributing to climate change (IPCC, 2014). Hence, without a change towards a more sustainable energy supply and demand, no substantial green transformation seems possible. But the expansion of renewable energy raises ecological issues (e.g., regarding landscapes) and social issues (e.g., access of the poor to energy services).

#### 3.2.2 Achievements so far related to the energy sector

In the last few years, Vietnam has been very active in developing policies and projects for green transformations in relation to energy issues. Vietnam has been one of the major beneficiaries of the Clean Development Mechanism (CDM), achieving total GHG emission reductions of about 137.4 million CO_2_ emissions equivalents by 2015. Nearly 90% of the over 250 registered CDM projects were in the energy sector. Energy plays also a key role in Vietnam's Nationally Appropriate Mitigation Actions (NAMAs; NAMA Database, 2017). Nevertheless, GHG emissions in Vietnam are still very high with an increase of 878% in the period 1990–2014. This is the highest value in the ASEAN region (World Resources Institute, 2015). Vietnam has a good potential for the development of renewable energy, such as wind, solar, biomass, and hydropower (geothermal is under consideration). Vietnam increased its share of hydropower, as well as wind and solar energy generation in recent years as showed in Figure 5. Old coal‐fired power stations are being replaced by more modern and less polluting gas turbines and renewable energy. Government officials suggest that Vietnam could replace as much as 8 GW of the coal‐based electricity plants with renewable energy plants by 2030. Wind energy has a high potential in Vietnam, and the first wind farm was completed in Binh Thuan province in 2009. The government estimates that over 500 MW of wind energy could be generated in Vietnam (Dang, 2016). As of 2017, the total installed capacity was estimated as nearly 190 MW for wind and over 7 MW for solar photovoltaic (DEVI Renewable Energy, 2017). The country also has a high potential for hydropower, both small scale and large scale due to an abundance of rivers and suitable topography. Already, today, over 160 MW of small‐scale hydropower capacity is installed. Hydropower accounts for nearly 7% of the total current energy supply of Vietnam (IEA, 2017).

Hydropower relies mainly on small‐scale hydropower dams. However, our fieldwork finds that the hydropower potential has already been almost exploited. Moreover, due to the social and environmental implications of hydropower development, the government has decided to cancel several new projects, for example, around 300 new projects were cancelled in 2012 due to environmental concern and protests from NGOs and local people. In Lao Cai province, 125 projects were presented for development to the government; however, only 75 projects have been approved.

**Figure 2:**
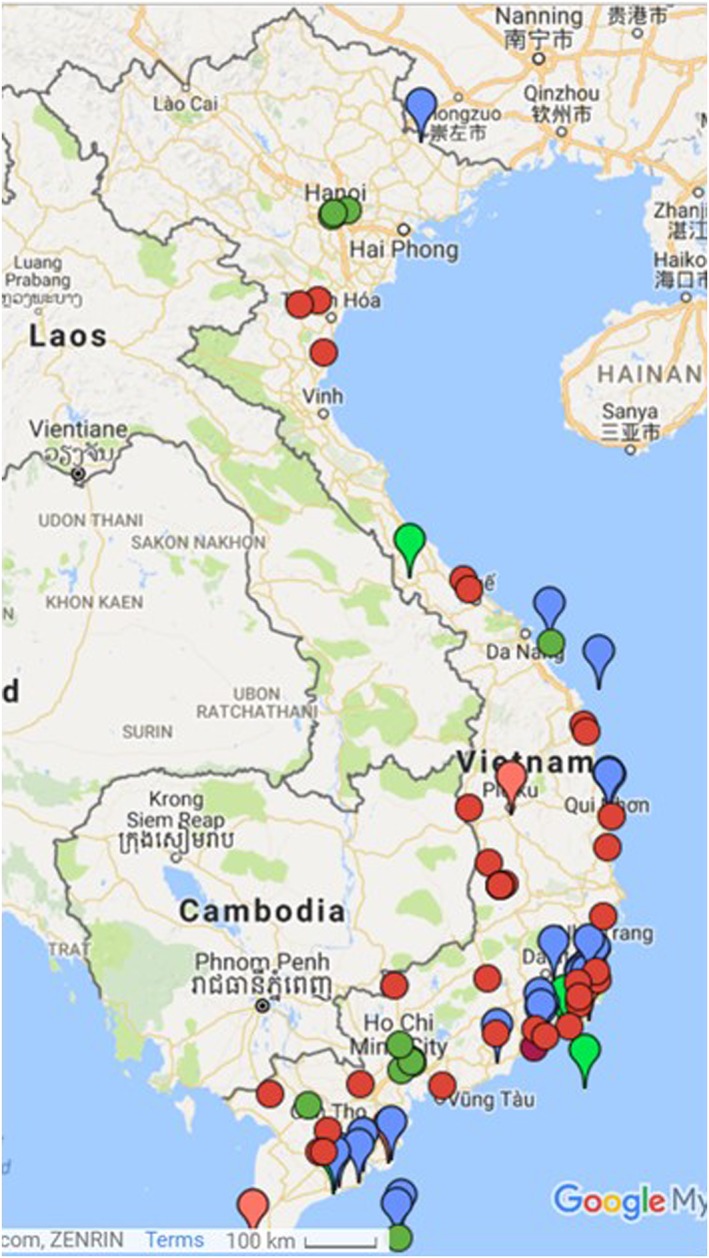
Map of solar and wind projects in Vietnam. Green dot: solar project in operation; red dot: solar project under construction or in pipeline. Green drop: wind project in operation; blue/red drop: wind project under construction or in pipeline. Google My Maps, based on information from DEVI Renewable Energy (2017)

There is high potential for wind farm development. There are currently three pilot projects in the centre/south of the country and in the islands. Moreover off‐shore projects are under consideration/study. There are various social and environmental issues linked to wind energy: (a) noise, (b) impacts on birds' migration, and (c) land occupation and compensation issues, yet most interviewees found the implications of wind energy rather minor in Vietnam, particularly in comparison with the implications of hydropower projects. DEVI Renewable Energy (2017) suggests that five wind farms are currently operating in Vietnam, and another 29 are currently under construction or in the pipeline. The source also indicates that 12 solar parks are currently in operation, and another 31 are under construction or in the pipeline. See Figure 2. These maps need to be treated as a work in progress as over the years, new wind and solar projects will be constructed, some will not be constructed despite being in the pipeline due to financial and planning challenges, and others will be delayed due to various reasons.

Solar energy is mainly used for heat purposes (i.e., solar water heaters). Big solar projects are being investigated as part of feasibility studies, but there are no on‐going projects at the moment. There is a road map for PV solar development with several pilot projects that are under construction or planned, such as the 19.2‐MW Thien Tan solar power project located in the central province of Quang Nam's Mo Duc district, the 50‐MW Thien Tan Solar Energy Project in Bac Ai district in Ninh Thuan province, and the 100‐MW Mekong Delta's Long An province project (Vietnam Investment Review, 2017).

The fieldwork found that the main barriers to solar and wind energy development are as follows: (a) technology costs, (b) lack of local expertise, (c) internal tariffs and lack of financial incentives, and (d) need of financial investments from abroad. Renewable energy projects have feed‐in tariffs schemes, which make them more financially attractive. However, the tariffs are still not at a sufficient level to cover the risks and incentivise large‐scale private sector investments across all technologies (GGGI, 2017).

Biogas is produced in rural areas from both biomass and manure. There are already several small projects in rural areas and more are being developed. The GGGI is supporting a new project called Scaling up Biomass Waste‐to‐Energy in Vietnam. The project will explore possibilities for the transformation of agricultural waste products into energy by working directly with farmers to provide additional sources of income (GGGI, 2017). Electricity production from biomass is expected to reach 2.1% of the total electricity produced in 2030 in Vietnam (GIZ, 2017). However, due to the high increase of energy demand in Vietnam, energy from coal will also be an important share of the total energy produced. During 2005–2014, the average annual growth of electricity demand was 12.1%, and due to rapid economic growth, electricity demand is expected to increase in the future (ADB, 2015).

**Figure 3:**
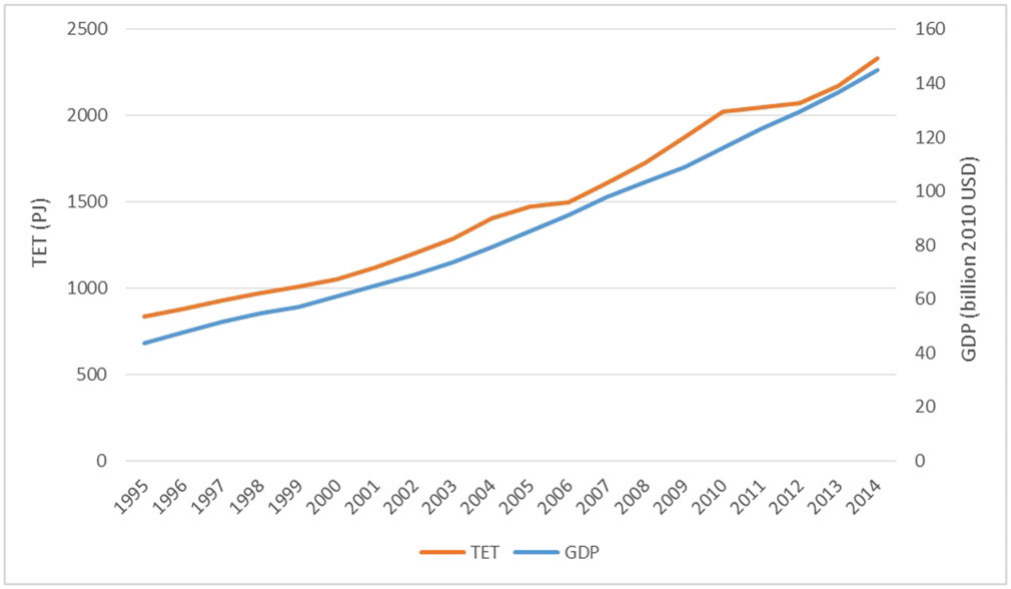
Total energy throughput (TET, total final energy consumption) and GDP in Vietnam between 1995 and 2014. Calculated by the authors based on data from the IEA (2017)

**Figure 4:**
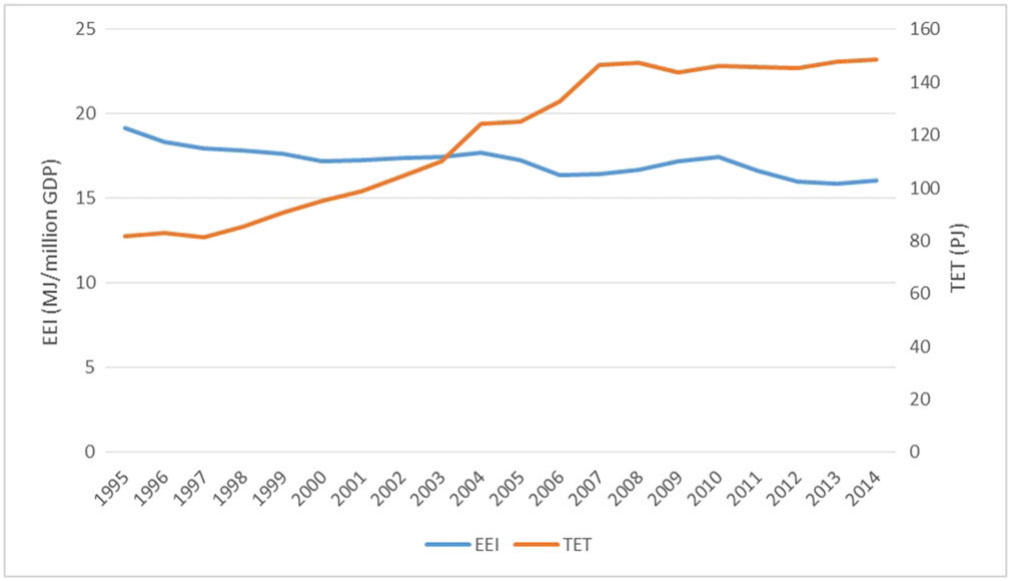
Energy efficiency indicator (EEI, energy consumption per million GDP) and total energy throughput (TET, total final energy consumption) in Vietnam between 1995 and 2014. Calculated by the authors based on data from the IEA (2017)

The following figures show key energy data for Vietnam. Figure 3 indicates the total final energy consumption in comparison with the Gross Domestic Product (GDP), and Figure 4 shows the energy consumption per million GDP in comparison with the total final energy consumption. GDP and energy demand have increased steadily in the last years (Figure 3), and projections on energy demand indicate that in 2035, the total final energy demand will be 2.5 times higher than in 2015 (Danish Energy Agency, 2017), posing serious challenges on GHG emissions reduction.

**Figure 5:**
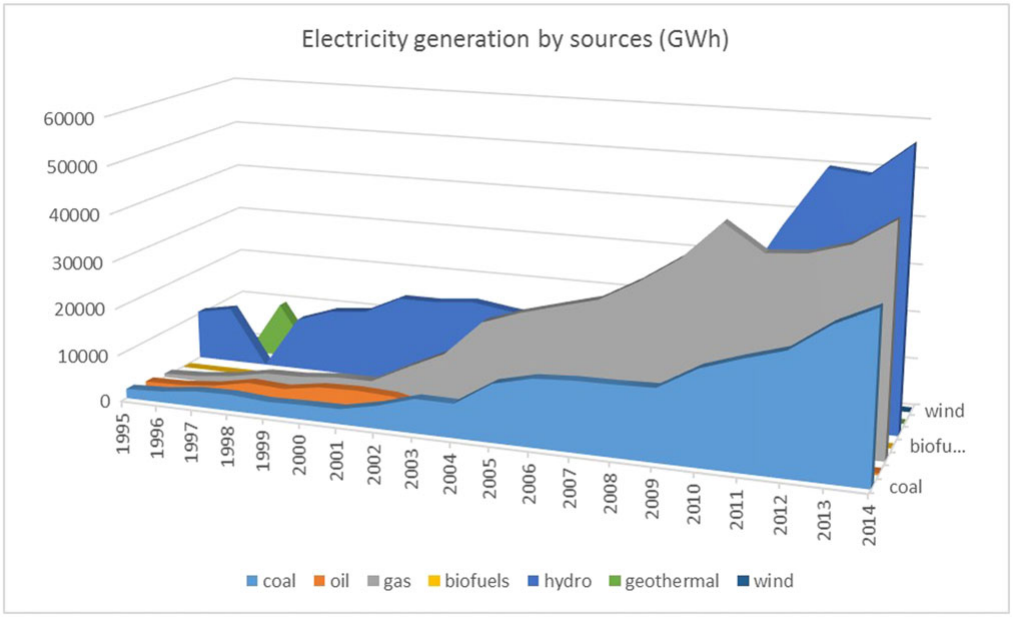
Electricity generation from renewables in Vietnam between 1995 and 2014. Data from IEA (2017)

Figure 5 indicates the overall electricity generation from various sources, which is mainly from hydropower followed by gas and coal. Although the electricity generation from nonrenewable sources has been growing steadily over the last few decades, the share of renewable energy, particularly hydropower, has increased steadily too. Electricity generation from wind has also increased from 1 GWh in 2008 to 87 GWh in 2014. Solar PV–installed capacity is expected to increase from around 6 to 7 MW by the end of 2015 to 850 MW by 2020 (1.6% of the country's power generation) and 12,000 GW by 2030 (equivalent to 3.3%) (GIZ, 2017). Moreover, Although the share of electricity productions of wind and solar is expected to increase from 0.8% in 2020 to 2.1% in 2030 for wind and from 0.5% to 3.3% for solar, hydropower will decrease from 29.5% to 15.5%, indicating a willingness to shift towards less risky investments in terms of climate change, social resistance, and geopolitical tensions, as almost 60% of Vietnam's rivers originate from outside of its borders (GGGI, 2017; GIZ, 2017; Urban, Siciliano, & Nordensvard, 2017).

**Figure 6:**
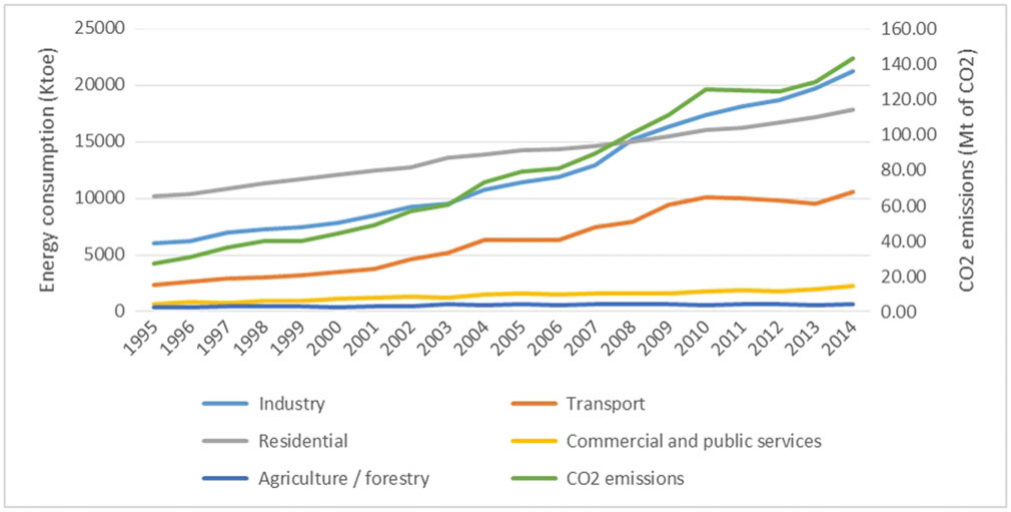
Energy consumption and CO_2_ emissions by sectors in Vietnam. Data from IEA (2017).

Figure 6 displays the energy consumption and CO_2_ emissions by sectors in Vietnam. The most energy‐consuming sectors are the industrial sector, followed by the residential sector, and the transport sector.

### 3.3 The role of the state

The state and its agencies are important drivers for promoting and implementing green transformations, including in the global South (Lederer et al., 2018; Lederer et al., Forthcoming). This is particularly the case for Vietnam, which has a strong government that aims to drive green transformations in the energy sector as well as in the agriculture and forestry sector. Schmitz, Dau, Pham, and McCulloch (2015) also argue that the Vietnamese government is working closely together with the private sector, both national and foreign enterprises, to achieve economic development.

The Vietnamese government plays a key role for shifting its strategic approach from climate change adaptation to mitigation as a way to achieve economic restructuring, enable energy security, and leverage new finance and access to technology in a time when traditional development aid comes to an end. Renewable energy is therefore seen as a national priority by policymakers. The government provides import tax exemptions and land fee exemptions for those investing in renewable energy.

Interviewees claim that participation and inclusive development is a successful story in Vietnam with the integration of top‐down and bottom‐up approaches. For instance, the draft GGGI Vietnam Country Planning Framework (CPF) for the period 2016–2020 is the result of intensive consultations held over the course of 2015 with ministries, civil society (i.e., the Green Growth Civil Society Organization), agencies, donors, and development partners specialized in green growth. The consultation process has been based on a series of bilateral meetings with civil society, the private sector, and government to formulate ideas, as well as donor roundtable and a final workshop to present an early draft to the Ministry of Planning and Investment (MPI) and other governmental representatives. Similarly for the preparation of master plans for cities, GGGI has to follow a strict consultation process through the organisation of workshops to formulate strategies and ideas involving the local populations, the provincial, and cities' peoples committees (interview with representative of GGGI, October 10, 2016).

Yet there is a high control from the government on means of communication and media and low transparency of laws. In terms of protests, people are not allowed to protest on the streets, but only in front of offices. Protests must be apolitical as the interviewees report. Hence, overall, there are still strong boundaries with view to the democratic quality of domestic politics, given the tough stances on freedom of speech, freedom of press, and freedom to assemble.

### 3.4 Energy justice

Three major findings of the fieldwork are as follows, see also Table 2 for a summary of the main issues. First of all, energy security is at the forefront of drivers for green transformations in Vietnam, which also relates to the public acceptance of the political system. Green transformations are in line with strategic decisions to restructure Vietnam's economy, increase its competitiveness, and create employments, as well as improving energy security and promoting national stability and well‐being. This is indicated in the following quote:

The government plans until 2030 offer new opportunities and advantages at wider‐economy level. This includes industrial policies for a green future, following what Korea or China have done. This creates opportunities for economic growth and it is driven at high‐level. Green transformations are an opportunity to avoid black‐outs and brown‐outs, there are opportunities for energy access, particularly in the Mekong Delta. You know, the Mekong Delta is very energy‐consuming due to Hoh Chi Minh City and there are only weak transmission lines between the North and South. Vietnam is building power plants, but they are not ready yet. The security of energy supply for industries and consumers is very important. It links to the public acceptance of the political system, to public happiness. There is a need to grow the renewable energy sector to improve the security of supply. So far renewables are only a small percentage, they are not big enough yet, but high‐level policy‐makers see the green growth strategy and the renewable energy expansion as important for national development and important for the energy security of supply. (Interview with representative from ADB, October 5, 2016)

**Table 2:** Energy justice framework for green transformations in Vietnam's energy sector

Procedural justice		Distributional justice	
Availability	99% electricity access	Availability	Urban areas: 100% electricity access, rural areas: 98% electricity access. No information about other distribution issues such as energy access for female‐ vs. male‐headed households, young vs. old etc.
Affordability	Government controls electricity prices. Electricity price hikes in recent years to recover losses of SOE Vietnam Electricity Group (EVN) that controls electricity generation, transmission, distribution, allocation, retail, and import.	Affordability	Different rates of electricity for various uses (households, industry, and agriculture). The more electricity is being used, the more expensive it becomes; hence, the policy is more favourable for low energy users on lower incomes.
Transparency and accountability	Interviewees claim energy plans and decisions are made transparent by various government agencies. Accountability desired to appease local population, yet limited due to Socialist state, absence of democratic elections.	Transparency and accountability	Variable depending on each energy project, yet interviewees claim the government aims for transparency due to central government plans and accountability of local governments.
Intragenerational and intergenerational equity	GHG emission reductions GHG emissions target: 8% by 2030 compared to BAU, emission intensity reduction 20% by 2030 compared to 2010 levels. If bilateral and multilateral financial and technical support will be made available to Vietnam under the Paris Climate Agreement, the target can be increased to 25% GHG emission reductions and 30% emission intensity reduction by 2030. Closure of coal‐fired power stations, fewer new coal‐fired power plants built.	Intragenerational and intergenerational equity	Reduction in GHG emissions and emissions intensity now for the benefit of future generations. Lower reliance on coal‐fired power plants to reduce emissions for future generations.
Responsibility	Interviewees report that project consultants / power producers are held responsible by local government, which is held responsible by national government. Policies and national plans embed the responsibility to protect the natural environment and reducing energy‐related environmental adverse effects.	Responsibility	Variable depending on each energy project, yet structuralresponsibilities embedded between national government, local government and power producers/project consultants. Today's generation seems to be more aware of the responsibility to protect the natural environment and reducing energy‐related environmental adverse effects, also for future generations.
Due process	Interviewees report high level of due process, EIA legislation in place that is usually followed. Government‐fixed compensation rules.	Due process	Interviewees report high level of due process, EIA legislation in place that is usually followed. Difference may exist between various energy projects. Local people usually compensated for adverse effects stemming from energy projects according to government‐fixed compensation rules.
Sustainability	Government plans aim for sustainable development and green growth, by increasing the share of renewable energy among total electricity generation, increasing energy efficiency, introduction of CDMs, NAMAs, INDC, REDD+.	Sustainability	Government plans aim to balance environmental, social, and economic sustainability.
Resistance	Interviewees report options for complaints and grievances.	Resistance	Interviews claim energy projects usually have procedures for complains and grievances.
Intersectionality	Cross‐cutting attempts to reduce social injustice, climate injustice, and energy injustice reported by interviewees.	Intersectionality	Cross‐cutting attempts to reduce social injustice, climate injustice, and energy injustice, particularly for the poorest, according to the interviewees.
Recognition	Energy access and energy security agenda is strongly represented, according to the interviewees.	Recognition	Aims to increase energy access in rural areas, improve energy security to reduce the occurrence of black‐outs and brown‐outs for individual consumers and industries in specific.
Distribution	Attempts by the government and project developers to distribute the costs and benefits of energy generation equally, according to the interviewees.	Distribution	Costs and benefits of energy generation not evenly distributed regarding the impacts of dams on local people and the benefits elsewhere (e.g., electricity transported to cities). Uneven distribution of costs and benefits for coal miners due to health and safety issues. Otherwise relatively even distribution of costs and benefits.
Fair procedure	Aiming for fair procedures by having in place thorough legislations for EIAs/ESIAs, requests for community consultation and compensation.	Fair procedure	Variable depending on the implementation of each energy project, yet fair procedures in place in theory.
Restorative justice	Interviewees mention compensation payments for local people affected by energy projects, particularly hydropower, are set at government rates. Mitigation of social and environmental impacts advised by government agencies.	Restorative justice	Variable depending on the implementation of each energy project, yet fair procedures in place in theory for compensation and mitigation of social and environmental impacts, according to interviewees.

Moreover, intergenerational strategies to achieve green transformations in the energy sector are evident in national legislation, such as in Vietnam's Renewable Energy Development Strategy 2016–2030 (REDS) with outlook until 2050 and the Revised National Power Development Plan 2011–2030. These strategies have the aim to increase renewable energy generation among the country's energy mix in the long‐term and to diversify energy sources with the ultimate goal to limit CO_2_ and GHG emissions from the energy sector (IEA, 2016). This is indicated in the following quote:

… we can see a lot of those future coal fired power stations not come online and that would be a massive win for Vietnam, that would really show a shift in the business as usual scenario. We have seen it already in the revised national power development plan, there is a number of new coal fired power stations that are not there anymore, so we have already seen a shift. If Vietnam can keep going in the right direction then we can ensure there is less pollution to be cleaned up in the future.” (Interview with GGGI, October 10, 2016)

This refers to intergenerational justice in Vietnam's energy planning. Second, some responses from our interviews indicate that the government does not pursue the restructuring of the energy sector at all environmental or social costs. A series of hydropower dams were reported to have been cancelled due to social and environmental impacts. This is indicated in the following quote:

Key concerns regarding the energy sector are greenhouse gas emissions, impacts of energy infrastructure projects on local people and impacts on the environment, like hydropower projects flooding the natural environment and impacts on local people. These serious problems are being recognised by government and government think tanks. Some hydro projects—big dams—were cancelled due to environmental and social concerns, for example two large dam projects in central Vietnam. Many small dam projects were also taken out of energy plans due to adverse social and environmental impacts, so the projects were withdrawn. These dam projects are proposed by state‐owned or independent consultants, then proposed to local government, then submitted to central government. The central government rejects it due to social and environmental impacts. Sometimes central government agrees to these plans, then change their minds and cancel the projects. The National Assembly has the final say on decisions about dams. Sometimes they suggest modifications to certain plans, for example the National Assembly was presented with a plan for one big dam, but rather they suggested to build two smaller dams because of the impacts on forests, height of the dam wall and impacts on local people. The decisions are case by case. (Interview with representative of Institute of Energy Science, October 5, 2016)

Due diligence processes are reported to be followed, such as Environmental Impact Assessment (EIA) and Environmental and Social Impact Assessment (ESIA) procedures. This is suggested in the following quotes:

“EIA legislations and policies and regulations are followed to a high standard in Vietnam, better than in other developing countries” (Interview with representative from ADB, October 5, 2016). “The dam project was funded by the World Bank, so a lot of requirements needed to be met including a proper ESIA. We met all the requirements and very carefully followed all the procedures and standards” (Interview with representative of Coc San hydropower project, October 8, 2016)

Third, trade‐offs are still rather substantial with regard to hydropower projects and partly also with wind, solar, and biomass energy projects, if land needs to be appropriated from local people. It seems like there are efforts for following due processes, being transparent, and accountable to reduce some of the impacts of these trade‐offs. However, interviewees report that even for exemplary dam projects, land grabs can take place because the land belongs to the government:

We had to take land from the local people, but luckily no houses .... (Interview with representative of Coc San hydropower project, October 8, 2016)

The implementation of energy projects usually has a strong link to land use governance. A particular feature reported by interviewees is that there is no private land in Socialist Vietnam, instead the land is allocated to individuals by the state with land‐use titles of maximum 50 years with a certificate. Farmers have the possibility to sell the certificate to other farmers or companies. Land consolidation is an on‐going process in Vietnam through the establishment of cooperatives of farmers or the introduction of private companies. Problems occur particularly where there are unequal power relations (e.g. a big private company or a big state‐owned enterprise vs. local residents) and where best practices and due diligence processes are not followed.

For new hydropower projects, it is being reported that efforts are being made to compensate the local people affected by the projects and to incorporate elements of Corporate Social Responsibility (CSR) too. For example, interviewees reported that this has happened for the Coc San hydropower project, which is a small gravity dam with an installed capacity of 29 MW, 300 km away from Hanoi, in Lao Cai province. The dam was financed by a World Bank loan, built by Chinese contractors such as Zhejiang Hydropower Company, used Vietnamese, and OECD consultants, such as UK‐based ERM for doing the Environmental and Social Impact Assessment (ESIA); is partly Vietnamese state‐owned and partly owned by Singaporean investors; and is certified as a CDM project with a total of 75,000 CER/year set at a carbon price of 5US$ per CER.

Interviewees claim that land grabs from local people happened, but only in uninhabited areas; they reported that there were no relocations of people needed. Interviewees reported that a range of mitigation strategies are in place to address the adverse social and environmental impacts of the dam, including afforestation measures, agricultural loans available for local people, offering scholarships to local people, and monetary compensation paid to the 160 affected households set at government‐fixed rates. These are reported to be fixed compensation rates for lost crops, lost land, lost trees plus support to the locals to improve their incomes, essentially a form of living allowance. The project developer also claimed that there are procedures in place for grievances and complaints. The following quotes indicate that some of the CSR and compensation measures are said to be in place to deal with the negative trade‐offs of the dam:

We paid for a kindergarten and local school for the local people. There is monetary compensation for crops, trees, land and support money. (Interview with representative of Coc San hydropower project, October 8, 2016)

The interviewees also claim that there are opportunities for local employment creation, rural development, and access to electricity for the local population.

“200 people were employed in the dam construction, 30 were Chinese engineers brought from China, there were also some international construction workers from China and India, the rest were local people” (Interview with representative of Coc San hydropower project, October 8, 2016)

Further research will be needed to verify these claims, including interviews and focus group discussions with the local population, for which the research team did not get permissions from the local government at this point in time. Local people affected by the dam may be having different views on the socio‐economic impacts of the dam project and whether or not they felt adequately compensated. Previous research of some of the authors suggests that there is often a large gap between the perceived realities of local people affected by dams and the perceived realities of project developers, planners, and policymakers (Siciliano et al., 2018; Siciliano & Urban, 2017).

Land issues, resettlement, and compensation may be key issues for other large infrastructure projects too, including large on‐shore wind farms and large solar PV parks. Yet more research is needed into this issue. For other renewable energy projects, such as offshore wind energy and roof‐top solar PV, there may be less trade‐offs and concerns, as the following quote indicates:

There are projects on solar PV roof‐tops for government buildings, biomass energy projects, wind projects. In Vietnam the wind speed average is under 10 m/second. There are mainly small to medium turbines. Land occupancy is low, so the local impact is small, and there is a favouring of coastal and offshore wind turbines, which have even less impacts on social and environmental issues. For offshore wind projects there are no negative impacts on social issues. The positive impacts are employment creation and access to electricity for the local population.” (Interview with representative of Institute of Energy Science, October 5, 2016)

The issues explored above have been summarised in Table 2, where we apply the energy justice framework to green transformations in Vietnam as outlined in Table 1.

## 4 DISCUSSION AND CONCLUSION

This article addressed the opportunities, barriers, and trade‐offs for green transformations in Vietnam's energy sector and examines this from an energy justice perspective. The article draws on in‐depths expert interviews with representatives from government agencies, private firms, academic institutions, and multilateral institutions in Vietnam. The article also evaluated recent energy data for Vietnam.

Green transformations are particularly relevant for countries in the global South as they are in the process of development but faced with environmental limitations such as climate change and resource scarcity. Vietnam is actively pursuing opportunities for green transformations in the energy sector to follow three key targets: achieve green growth, sustainable development, and tackle climate change. Key opportunities for green transformations are embedded in the wider national development agenda such as promoting sustainable economic growth, increasing employment opportunities, providing energy access, and very importantly, increasing energy security by relying on domestic, renewable energy resources. Other opportunities are promoting energy efficiency and access to modern energy technologies and reducing environmental pollution. Vietnam has already benefited from an abundance of energy‐related CDM projects and has thereby reduced GHG emissions significantly. It also invests heavily in renewable energy, most importantly, wind power and hydropower, which already accounts for nearly 7% of the total primary energy supply (IEA, 2017). Green transformations are therefore well under way in Vietnam's energy sector.

Barriers to green transformations in Vietnam are (a) confusion about the overlapping strategies for green growth, sustainable development, and tackling climate change; (b) competing ways of implementing these three strategies as well as competing policies (e.g., policies that favour industrial development and economic growth vs. policies that favour environmental protection vs. policies that favour reduction of GHG emissions); (c) lack of coordination between experts and policies; (d) lack of a policy framework to attract investments in the field of green transformations; and (e) the roadmap for introducing renewable energy that is very modest and not ambitious enough.

Trade‐offs are mainly related to energy projects and land ownership. Cases of land grab have been confirmed, for example, for hydropower projects, as the government remains the owner of land in Socialist Vietnam. Also for other energy projects, including wind farms and solar parks, there are issues related to land, such as land availability, land ownership, and the competition between land for agriculture and land for energy generation. However, there are efforts for following due processes, being transparent, and accountable to reduce some of the impacts of these trade‐offs. Government‐fixed compensation rates are said to be applied to local people who loose land, crops, and trees due to energy infrastructure projects. Some projects offer additional CSR such as building schools and kindergartens for local affected people, as well as offering a living allowance. Yet whether these measures meet the actual concerns of most people affected is a question that needs to be addressed in more specific future empirical studies. Other trade‐offs are related to deforestation, as forests sometimes need to be clearer for energy projects, for example, when a dam will be built and a forested area will be flooded for creating a reservoir. Down‐streams flooding, erosion, sedimentation, loss of biodiversity, habitat destruction, impacts on fish and other aquatic species are the direct impacts of hydropower, particularly of large dams.

The fieldwork indicates that government agencies seem to be concerned about factors relating to energy justice, particularly with regard to enabling energy security to avoid frequent black‐outs and brown‐outs and to protect industries and individual consumers as a way to enable public order. Keeping the energy supply stable may be considered a way to appease the masses and keep national stability and integrity. Intergenerational justice seems also a concern as the government makes revisions to existing plans, such as the Revised National Power Development Plan 2011–2030 to reduce the reliance on fossil fuels, increase the share of renewable energy, and lower GHG emissions for the benefit of today's and tomorrow's generations. The government seems also aware of the trade‐offs of energy projects on the local people and the local environment, even going so far as to cancel several dam projects due to their adverse social and environmental effects, as reported by the interviewees. This concern for the well‐being of local people and the local environment is crucial (yet rarely found in Southeast Asia) for enabling equitable, socially just green transformations. However, as the government is the owner of all land in Vietnam, land grabs still occur for energy projects, and while compensation mechanisms are in place, the land grabs can have devastating impacts on (poor) people, their way of life, and their livelihoods. More attention needs to be given to how these people cope and how to further support them.

In conclusion, this article identifies the following policy recommendations: It would be useful for the government of Vietnam to develop more coordinated, integrated approaches, policies and plans that span across the three areas that address green transformations: green growth, sustainable development, and climate change. A more holistic, integrated approach would enable better planning and implementation of strategies for green transformations that span across various sectors, perspectives, institutions, policies, and motives.

Adopting a more ambitious renewable energy strategy to increase the share of renewable energy, particularly wind and solar, would be another way to further promote green transformations. This could be linked to technology transfer and cooperation from developed countries under the framework of the Paris Agreement. The current INDC is staged for Vietnam, suggesting GHG reductions of 8% by 2030 compared with 2010 levels, which could be increased to 25% GHG emission reductions by 2030, if financial and technical support will be provided by the international community. This could be further negotiated either bilaterally with specific countries or multilaterally to gain access to climate‐relevant technology and financial support in exchange for increasing the share of renewable energy further. In conclusion, Vietnam is well on its way to promote green transformations in the energy sector. Yet there are various trade‐offs and energy justice concerns that have to be taken into account to enable green transformations to be sustainable opportunities for the country's future.
